# Somatic Acupoint Stimulation for Cancer-Related Sleep Disturbance: A Systematic Review of Randomized Controlled Trials

**DOI:** 10.1155/2020/2591320

**Published:** 2020-04-28

**Authors:** Xian-Liang Liu, Hui Lin Cheng, Simon Moss, Carol Chunfeng Wang, Catherine Turner, Jing-Yu Tan

**Affiliations:** ^1^Department of Nursing, Huazhong University of Science and Technology Shenzhen Union Hospital, 89 Tao Yuan Road, Nanshan District, Shenzhen, China; ^2^School of Nursing, The Hong Kong Polytechnic University, Hung Hom, Kowloon, Hong Kong; ^3^College of Health and Human Sciences, Charles Darwin University, Ellengowan Drive, Darwin, NT 0909, Australia; ^4^School of Nursing and Midwifery, Edith Cowan University, 270 Joondalup Drive, Joondalup, WA 6027, Australia; ^5^College of Nursing and Midwifery, Charles Darwin University, Ellengowan Drive, Darwin, NT 0909, Australia; ^6^College of Nursing and Midwifery Brisbane Centre, Charles Darwin University, Level 11, 410 Ann Street, Brisbane, QLD 4000, Australia

## Abstract

**Aim:**

The aim of this systematic review was to analyze and synthesize available evidence for the effects of somatic acupoint stimulation (SAS) on cancer-related sleep disturbance in adults with cancer.

**Methods:**

Nine databases and four clinical trial registries were searched from their inception to July 2019 to identify potential articles and registered trials. Two authors independently extracted data and appraised the methodological quality of the included studies. The included studies could not be subjected to meta-analysis due to the significant variations in SAS intervention protocols and outcome measurement instruments. This systematic review therefore reported the results of the included trials narratively.

**Results:**

Seven studies were identified, which involved 906 cancer patients. SAS protocols varied across trials without an optimal evidence-based standard intervention protocol to manage cancer-related sleep disturbance. Sanyinjiao (SP6) was the most commonly selected acupoint. Manual acupuncture was typically 15–30 min in duration and was conducted once a day or once a week for a period of 1–5 weeks, whereas self-administered acupressure was typically 1–3 min in duration per point and was conducted once a day, such as during night time before going to bed, for a period of 1–5 months. The results indicated that SAS could potentially relieve cancer-related sleep disturbance and improve quality of life. Mild adverse effects were reported in three of the included studies, but none of them performed a causality analysis to clarify the association between the reported adverse events and the intervention.

**Conclusions:**

This systematic review showed that SAS is a useful approach to relieving cancer-related sleep disturbance. However, research evidence on SAS for managing cancer-related sleep disturbance has not been fully conclusive due to the limited number of existing clinical studies with relatively small sample size and suboptimal methodological quality. Clinical trials with large sample size and robust methodology are warranted in future research.

## 1. Introduction

Sleep disturbance is one of the most frequently reported symptoms experienced by individuals affected by cancer, with a prevalence ranging from 14% to 93% [1–3]. Cancer-related sleep disturbance is manifested as early morning awakening, trouble falling asleep, and difficulty staying asleep [4]. Cancer-related symptoms, such as anxiety, depression, fatigue, and pain, tend to be exacerbated by sleep disturbance [5]. Sleep disturbance can cause significant psychological distress in patients with cancer [6]. Sleep disturbance also appreciably impairs the functional status and quality of life (QoL) of patients with cancer [7]. Cancer-related sleep disturbance has become an important health issue, which can impair patient outcomes; however, this problem has not yet been adequately addressed in health care settings [8].

Somatic acupoint stimulation (SAS) is a broad term that describes body acupoint stimulation methods, with manual acupuncture and acupressure, to balance the excess or deficiency of *Qi* on the basis of traditional Chinese medicine (TCM) theory [9]. Manual acupuncture entails needle insertion into the body at specific acupoints, whereas manual acupressure refers to the stimulation of acupoints by applying pressure using the fingers or thumbs [10]. Multiple systematic reviews reported that SAS is a safe and effective therapy in managing sleep disturbance in noncancerous populations [11–15]. Patients with cancer would also prefer SAS to manage sleep disturbance given that this approach elicits fewer side effects than other pharmacological treatment options [15, 16]. However, effects of SAS on cancer-related sleep disturbance have not been fully conclusive in current clinical pracrtice and research.

Clinical studies have examined the effects of SAS on cancer-related sleep disturbance with contradictory findings reported[17–22]. Several small scale clinical trials (sample size ranged from 10 to 94) reported that SAS can alleviate sleep disturbance in patients with cancer [18–21], whereas another three-arm clinical trial with 288 cancer patients showed no significant differences among the effects of relaxing acupressure, stimulating acupressure, and control treatment on the sleep quality of patients with cancer at week 10; only the relaxing acupressure group reported significantly better sleep quality than the control group at week 6 [22].

One recent systematic review that evaluated the effects of acupuncture on insomnia in patients with cancer showed that acupuncture might be superior to conventional medications and sham acupuncture for managing cancer-related sleep disturbance [23]. However, this review excluded acupressure as one of the most important and commonly practised modalities of SAS, and included a small number of clinical trials with limited sample size [23], all of which contributed to inconclusive evidence on SAS for managing cancer-related sleep disturbance. Currently, researchers have not reached consensus on the optimal acupoint formula or intervention dosage to apply SAS to manage cancer-related sleep disturbance [19, 22]. Whether the existing evidence is scientifically rigorous enough to support the use of SAS for cancer-related sleep disturbance management remains unclear.

This systematic review was therefore designed to further explore the role and safety of SAS, including both body acupuncture and body acupressure, for the alleviation of cancer-related sleep disturbance. Specifically, this review explored (1) the best available evidence for the effects of SAS on cancer-related sleep disturbance; (2) the commonly used SAS intervention protocols to manage cancer-related sleep disturbance; (3) the commonly utilized research instruments to assess cancer-related sleep quality; and (4) the safety and cost-effectiveness of using SAS for managing sleep disturbance in patients with cancer.

## 2. Methods

### 2.1. Study Selection

The Preferred Reporting Items for Systematic Reviews and Meta-Analyses (PRISMA) checklist [24] was used in determining the content required and systematic process for this systematic review. The protocol of this systematic review has been registered at PROSPERO (no. CRD42019131609).

Nine databases were searched from their inception to July 2019 for the identification of potential articles and registered trials published in English and Chinese. The databases were Allied and Complementary Medicine, Cochrane Central Register of Controlled Trials, Cumulative Index to Nursing and Allied Health Literature, Excerpta Medica Database, PsycINFO, PubMed, Web of Sciences, Chinese Medical Literature Database, and Wanfang Database. The following trial registeries for ongoing or completed trials were also searched: World Health Organization International Clinical Trials Registry Platform (https://www.who.int/ictrp/trial_reg/en/), ClinicalTrials.gov (https://clinicaltrials.gov/), National Research Register via the Department of Health, UK (http://www.dh.gov.uk), and the Australian New Zealand Clinical Trials Registry (http://www.anzctr.org.au/).

The search strategy examples are presented in Table 1 and the following MESH terms and keywords were used in developing electronic search strategies: *English*: “acupunctur^∗^”, “acupressur^∗^”, “acupoint^∗^”, “massag^∗^” “sleep”, “insomnia”, “neoplasms”, “tumour”, “cancer”, “malignan^∗^” “carcinoma^∗^”, and “oncolog^∗^”. *Chinese*: “针^∗^”, “穴位^∗^”, “睡眠障碍”, “失眠”, “睡眠”, “肿瘤”, and “癌症”. This systematic review developed an individualized search strategy for each database and screened all the reference lists of previous reviews and included studies for additional relevant articles.

Two authors (X.L. Liu and H.L. Cheng) independently assessed the titles and abstracts of all records identified through the broad initial search. All duplicated records were managed and subsequently excluded using EndNote X9. If the detailed information from the titles and abstract was unclear to the reviewers, the full text of the publications was retrieved. The final decision to include or exclude a trial was based on an assessment of the full texts of all retrieved publications and registered trials. Any disagreements during these processes were settled by discussion with a third author if necessary. An assessment Excel form detailing specific reasons for the exclusion of publications was developed.

### 2.2. Inclusion Criteria

*(1) Types of Study*. Randomized controlled trials (RCTs). *(2) Participants*. Adult patients with cancer reporting sleep disturbance regardless of types or stages of cancer or previous cancer treatments. *(3) Intervention(s)*. SAS: manual acupuncture or manual acupressure on the specific body acupoints as interventions performed or taught by acupuncture practitioners, Chinese medicine practitioners, nurses, or other health care professionals. All forms of auricular acupoint stimulation, electronic acupoint stimulation, point injection, and transcutaneous electrical nerve stimulation were excluded. *(4) Control*. Routine methods of treatment for cancer-related sleep disturbance, sham (placebo) SAS, or usual care. *(5) Primary Outcomes*. (a). Cancer-related sleep disturbance and sleep quality measured by any validated tool and (b). self-reported hours of sleep and sleep efficiency, determined by the ratio of the total sleep time to the time spent in bed. *(6) Secondary Outcomes*. (a). QoL; (b). satisfaction with SAS; (c). safety of SAS, including any adverse events of SAS, such as dizziness and local bleeding, and the number of participants dropping out due to adverse events; and (d). cost-effectiveness of SAS.

### 2.3. Data Extraction

Two reviewers (X.L. Liu and H.L. Cheng) worked independently to extract the data of the included studies by using a predefined data extraction form. The research designs, study settings, patient demographics, SAS protocols (including the acupoint formula, intervention session, intervention duration and frequency), comparisons, and outcomes were extracted. If some data of the included studies were missing or unclear, the reviewers contacted the original authors of the included studies to seek clarification.

### 2.4. Methodological Quality and Risk of Bias Assessment

The risk of bias assessment tool, developed by the Cochrane Back Review Group, was applied to assess the methodological quality and risk of bias of the included studies as “high”, “low”, “unclear”, or “not applicable”. The tool comprises a set of specific questions related to randomization and concealment of intervention allocation; participant, health care provider, and outcome assessor blinding; cointervention avoidance; compliance; dropout rate; outcome assessment timing; selective reporting; and group similarity [25]. Two reviewers (X.L. Liu and H.L. Cheng) independently assessed the methodological quality and risk of bias of the included RCTs. Further details were identified by contacting the primary authors if the data were unavailable in the relevant reports. Any discrepancies between the reviewers were reconciled through discussion or consultation with a third reviewer.

### 2.5. Strategies for Data Synthesis

Only descriptive analysis, rather than meta-analysis, was conducted in this systematic review because of the considerable clinical heterogeneity identified in the SAS protocols, intervention dosages, time points of data collections, and measures of study outcomes. Meta-analysis has been deemed inappropriate for use in systematic reviews with substantial clinical heterogeneity of the included studies [26]. Clinical heterogeneity generally refers to variability in the study participants, intervention characteristics, and types or time points of outcome assessment [27]. In meta-analysis, credibility of the quantitative synthesis findings can be significantly affected by the clinical heterogeneity of the analyzed studies, often skewing the conclusions [27, 28]. Funnel plot and Egger's bias test were not performed to gauge potential publication bias in this systematic review given that only seven trials were included.

## 3. Results

### 3.1. Selection of Studies

**Figure 1:**
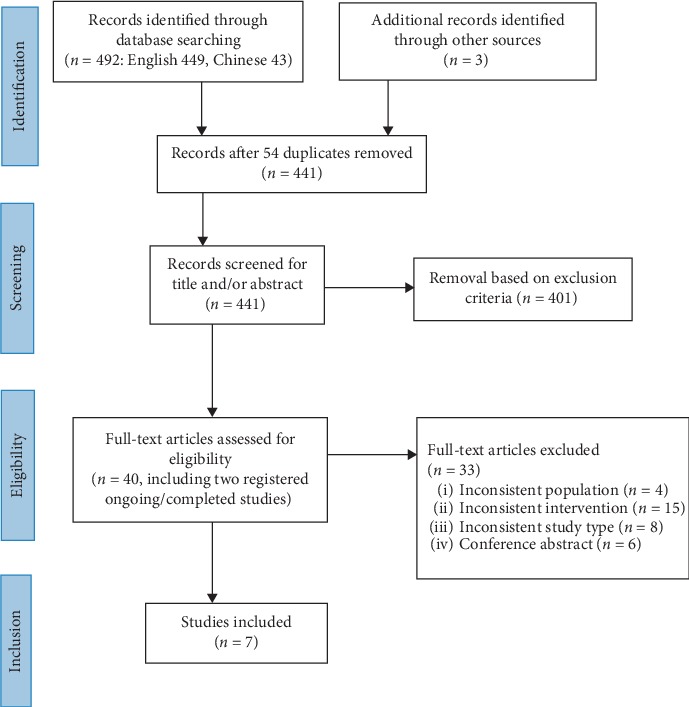
PRISMA flow diagram for search results.

Figure 1 illustrates the PRISMA flow diagram of information through the review. This diagram specifies the number of records identified at each stage and the reasons that records were removed. The title and abstract of 495 potential records were reviewed. Following the inclusion and exclusion criteria, 40 abstracts appeared relevant to the research questions were considered suitable for further evaluation of their full-texts. Then, 33 articles were rejected at this stage because they did not meet the eligibility criteria. Thus, seven studies, including five published studies [19, 21, 22, 29, 30], and two registered ongoing or completed trials [31, 32], were finally included in this systematic review.

### 3.2. Characteristics of the Included Trials

Table 2 provides a summary of the seven studies included in this systematic review. The included studies comprised 906 randomized cancer patients. Only published articles (*n* = 5) reported the number of patients who completed the intervention. The average sample size of the included trials was 129 (range: 57–288). Six published studies or registered trials were published in English [19, 21, 22, 30–32], and only one study was published in Chinese [29]. The included trials were conducted in Mainland China [21, 29], the United States [22, 31], Denmark [19], Taiwan [30], and Vietnam [32].

Three studies comprised two arms [21, 29, 31], and the other studies comprised three arms. For control groups, placebo (sham) group was used in four trials [19, 30–32]. Four studies targeted a single cancer type, such as lung cancer [30] or breast cancer [19, 22, 31], whereas three studies recruited patients with different types of cancer [21, 29, 32]. The average age of participants in the included trials was above 50 years old [19, 22, 29, 30]. Three studies recruited only female patients with cancer [19, 22, 31].

The commonly utilized research instruments to assess cancer-related sleep was the Pittsburgh Sleep Quality Index (PSQI), which was used in four trials [21, 22, 29, 30]. Two types of assessment instruments were used to assess sleep disturbance and sleep quality in the included studies. The first type of instrument comprises qualitative assessment tools, such as sleep diaries, which were used to evaluate total sleep time (min), total time in bed (min), the number of night awakenings after sleep onset, and sleep efficiency [32]. The other type includes self-reported instruments, such as the Athens Insomnia Scale (AIS), the Insomnia Severity Index (ISI), and the PSQI [21, 22, 29, 30], which were used to quantify patients' sleep quality.

### 3.3. Somatic Acupoint Stimulation Protocols

Two types of SAS protocols were used in the included trials: (1) self-administered acupressure [22, 30, 32] and (2) manual needle acupuncture [19, 21, 29, 31]. In one needle acupuncture study, moxibustion was additionally used on two points: Shenque (CV8) and Guangyuan (CV4) [29]. Sanyinjiao (SP6) was used in five trials [19, 21, 22, 29, 30]. Yintang (EX-HN3) and Baihui (DU/GV20) were used in four; Shenmen (HT7), Zusanli (ST36), Taichong (LR3), and Neiguan (PC6) were used in three; and Taixi (KI3) and Hegu (LI4) were used in two. One registered trial did not report the selected acupoints [31].

For the duration of SAS, manual acupuncture was typically 15–30 min in duration and was conducted once a day or once a week for a period of 1–5 weeks, whereas self-administered acupressure was typically 1–3 min in duration per point and was conducted once a day, such as during night time before going to bed, from 1 to 5 months. For manual acupuncture, the depth of needle insertion was 0.5–1.5 cun [29]. Participants' self-acupressure skills regarding acupoint location, acupressure intensity, and stimulation techniques were taught by nurses who were trained by the main researcher and a TCM professional (the intervention protocol had been validated by six Chinese medicine experts) [32], by acupressure educators who were trained by the study investigator [acupuncturist] [22], or by research assistants (RAs) who were trained by qualified TCM practitioners [30]. One study used acupressure with oil as an intervention [30].

The full details of the SAS protocols used in the included studies are presented in Table 3.

### 3.4. Methodological Quality and Risk of Bias of the Included Trials

Table 4 presents the methodological quality and risk of bias summary of each included trial. Randomization was mentioned in all seven RCTs, and six of which provided the precise descriptions of the processes used to generate random sequences. Adequate allocation concealmentwas described in four RCTs (low risk of bias). Only five RCTs reported a blind design for the participants and outcome assessor (low risk of bias).

Common methodological issues identified across these trials included limited information on the method of randomization, allocation concealment, cointerventions, and study results. For example, limited information was used in the assessment of the methodological quality of two registered studies because the full-text publications of these two trials could not be found or have not been published yet [31, 32]. Five items were not applicable to the two registered studies [31, 32]. Only three published trials mentioned and used intention-to-treat analysis [22, 29, 30]. Funnel plot analysis was not feasible because of the scarcity of trials in this systematic review.

### 3.5. Descriptive Analysis of Outcomes

The included studies could not be subjected to meta-analysis due to the significant variations in SAS intervention protocols and outcome measurement instruments. Thus, this systematic review reported results from these included trials narratively.

#### 3.5.1. Pittsburgh Sleep Quality Index

Four of the included trials reported PSQI scores [21, 22, 29, 30]. Two studies reported a significant between-group difference in PSQI scores at the end of the study [21, 29]. In a three-arm RCT, Tang et al. reported that PSQI scores in the acupuncture with oil and acupuncture-only groups reduced compared with that in the control group [30]. The adjusted PSQI score in the acupressure with oil group changed significantly compared with that in the control group at day one of the third chemotherapy cycle [30]. The change in the mean PSQI score of the acupressure-only group was not as pronounced as that of the control group at day one of the sixth chemotherapy cycle [30].

Another three-arm trial reported an improvement in the PSQI scores in the two intervention groups—relaxing acupressure and stimulating acupressure—when compared with the control group but the difference did not reach statistical significance [22]. This study [22] reported that the PSQI scores at week six in the relaxing acupressure group were significantly lower than PSQI scores in the control treatment but were not significantly different from that in the stimulating acupressure group. At week 10, no significant difference between the three arms was observed [22].

#### 3.5.2. Disturbed Night Sleep

Only one of the included studies with a three-arm design used disturbed night sleep (rated as “yes” or “no”) as a sleep quality index [19]. After 5 weeks of acupuncture intervention, the disturbed night sleep of patients with breast cancer receiving acupuncture treatment showed a statistically significant improvement compared with those receiving sham acupuncture or no treatment [19]. The intervention effects on disturbed night sleep lasted for at least 12 weeks after the final acupuncture session [19].

#### 3.5.3. Athens Insomnia Scale

Only one of the included studies used the AIS to assess patients' baseline condition but did not report the post-intervention AIS data [29].

#### 3.5.4. Insomnia Severity Index, Sleep Parameter in Sleep Diary and Insomnia Reduction

Two ongoing or completed registered trials used the ISI [31, 32], and one utilized the sleep parameter [32].

#### 3.5.5. Quality of Life (QoL)

Two of the included trials reported data on QoL, which were measured by the Long-Term Quality of Life Instrument (LTQL) [22] and MOS-SF36 [26]. In a three-arm RCT, Zick et al. reported that, at week 6 and week 10, three of the four QoL subscales, including somatic, fitness, and social support, improved more in the relaxing acupressure group than the control group [22]. No significant improvement in QoL scores between the stimulating acupressure group and the control group for any subscale at either time point was observed [22]. Zhao's study [29] did not find any significant difference in QoL scores between the intervention and control groups.

Two ongoing or completed registered trials evalauted QoL, which was measured by the Functional Assessment of Cancer Therapy-General scale [32] and the Functional Assessment of Chronic Illness Therapy-Fatigue subscale [31]. Results for these outcomes have not been reported yet [31, 32].

#### 3.5.6. Adverse Events

Adverse events or safety related to SAS were mentioned in three trials [19, 22, 29]. One trial reported that four women experienced mild and temporary adverse events, and one woman experienced mild but long-lasting adverse events in the acupuncture group [19]. Zick et al. reported that six nonserious cases were related to acupressure treatment, and all adverse events were mild bruising at acupressure sites [22]. Another study reported that no adverse events were found in the acupuncture and control groups, and no participants dropped out of the study because of adverse events [29]. Zick et al. reported that one participant dropped out due to the adverse effects of acupressure: bruising [22]. No included trials performed causality analysis between the reported adverse events and the intervention.

#### 3.5.7. Satisfaction with Treatment

No included trials reported participants' satisfaction with treatment.

#### 3.5.8. Cost-Effectiveness

No included trials reported on costs of SAS.

## 4. Discussion

This systematic review included five published studies and two registered ongoing or completed clinical trials. Results of the five published studies showed that SAS could potentially relieve cancer-related sleep disturbance and improve QoL of patients with cancer. However, none of the included RCTs can be used in the meta-analysis because they utilized different intervention protocols and measurement instruments (indicating substantial clinical heterogeneity) or because they did not include the data of their results. Methodological quality of the included studies was identified to be suboptimal. The duration of follow-up was short in most of the included studies, and only one study reported a 6-month follow-up after the completion of acupuncture [31]. Large-scale and well-designed clinical trials with long-term follow-up are warranted to further explore the effects of SAS on cancer-related sleep disturbance

One relevant systematic review summarized RCTs on acupuncture for cancer-related insomnia, and two of its analyzed trials were included in our systematic review as well [23]. We excluded electroacupuncture and auricular acupuncture studies that were originally included in the review by Choi et al. [23], and included acupressure, an important type of SAS that is frequently used for cancer symptom management, in this review to comprehensively explore the current evidence on SAS for managing cancer-related sleep disturbance. The prior published review concluded that, on the basis of a low level of evidence, acupuncture might be more effective in reducing cancer-related insomnia than sham acupuncture, conventional medications, or hormone therapy [23]. Another relevant systematic review included 41 publications, three of which were related to acupuncture for cancer-related sleep disturbance, exhibited a high risk of bias, and reported positive outcomes [33]. The current review is consistent with previous reviews, indicating that SAS may be a useful approach to relieving cancer-related sleep disturbance. Nevertheless, recommending SAS for the management of sleep disturbance in patients with cancer may be premature due to the inconclusive evidence identifed through current literature.

By reviewing the RCTs included in this systematic review, we found that the SAS intervention protocols, including the selected acupoints, number of treatment sessions, duration of stimulation, and type of stimulation, varied significantly across studies. For instance, although some acupoints, such as Sanyinjiao (SP6), Yintang (EX-HN3), and Baihui (DU/GV20), were frequently used in several included studies, none of the included trials employed a standardized acupoint formula that higlights the main acupoints for managing cancer-related sleep disturbance. Manual acupuncture was typically 15–30 min in duration and was conducted once a day or once a week for a period of 1–5 weeks, whereas self-administered acupressure was typically conducted for 1–3 min per point once a day for a period of 1-5 months. However, none of the included studies investigated the “dosage effects” of the SAS intervention protocols, such as the effects of variations in the acupoint formula, intervention duration and frequency, on sleep quality in cancer patients.

Four trials compared true SAS interventions with sham (placebo) interventions [19, 30–32]. Two studies described the design of sham acupuncture or sham acupressure, and a similar approach was used for sham SAS which selected several nonacupoints in the same region as the true acupoints used in the intervention group [19, 30, 32]. Only one study highlighted that the selected sham acupoints were located away from meridians [19]. The sleep quality of participants in the true SAS intervention group significantly improved compared with that in the sham SAS group [19, 30] which supports the specific treatment effects of SAS. A further comparison between the sham SAS and no intervention was performed in only one study [19] which concluded no between-group difference. It was difficult to identify the size of the SAS placebo effects from the included studies given that only one small scale study reported statistical comparisons between the sham SAS and no treatment [19].

No serious adverse events were found in the included trials. SAS-related adverse events were mentioned in three trials, and all adverse effects were deemed to be mild [19, 22, 29]. Based on the limited information regarding the safety of SAS in the included studies, SAS could be used as a safe intervention for cancer symptom management with only mild adverse events occasionally reported. Some of the adverse events, such as mild bruising, can be effectively managed and prevented once proper SAS training and precautions are provided. SAS should be performed or taught only by a well-trained and qualified therapist, acupuncturist, or TCM practitioner. It should be noted that none of the included trials assessed the causality between the reported adverse events and the SAS, and some potential adverse events might be underreported given that most of the included studies recruited only a small number of participants.

## 5. Quality of the Evidence

Limited information can be used to assess the methodological quality of ongoing or completed registered trials [31, 32]. A successful design and implementation of blinding in SAS trials has been significantly challenging given that the treatment and control procedures are conspicuously different in nature. Considering that some outcomes, such as sleep parameters in a sleep diary and patient-reported questionnaires, were completed by the participants themselves, reporting bias is possible in the included studies. The long-term effects of SAS on cancer-related sleep disturbance have not been investigated. In addition, the sample sizes of the included trials are small and the effects of SAS may thus have been underestimated.

## 6. Implications for Further Research and Practice

The beneficial role of SAS in managing cancer-related sleep disturbance is promising; however, the evidence remains inconclusive given the limited quantity and quality of the included trials as well as the small sample size identifed in some of the trials. Two included studies primarily focused on the effects of SAS on cancer-related fatigue while sleep disturbance was only included as a secondary outcome [22, 30]. Clinical trials with large sample sizes and robust methodology are warranted in future research to further explore the effects of SAS on cancer-related sleep disturbance. Identifying the types of SAS and acupoint formula that work best for cancer-related sleep disturbance and the optimal SAS intervention frequency and duration are the key to develop an evidence-based SAS intervention protocol for managing cancer-related sleep disturbance.

Only four of the included studies used sham SAS as a comparative treatment, wherein some nonacupoints in the same region as the true acupoints used in the study intervention were selected [19, 30–32]. However, some of the nonacupoints might be located near the meridians, which might further generate some unwanted specific treatment effects on sleep problems and further undermine the causality assessment between the ture SAS and patient sleep outcomes. Appropriate sham SAS design is recommended in future research to distinguish the specific therapeutic effects of SAS from its non-specific placebo effects [34]. Locations of the selected nonacupoints in the sham SAS group should be away from meridians to avoid triggering specific treatment effects of acupoint stimulation. Future studies should also include a long-term follow-up to evaluate the long-term effects and safety of using SAS for cancer-related sleep disturbance management. Causality analysis should be performed between the reported adverse events and the intervention. SAS should be performed and taught only by well-trained practitioners to minimize potential adverse effects and unsafe practice.

## 7. Conclusion

This systematic review showed that SAS is a useful approach to relieving cancer-related sleep disturbance. However, research evidence on SAS for managing cancer-related sleep disturbance has not been fully conclusive due to the limited number of existing clinical studies with relatively small sample size and suboptimal methodological quality. Clinical trials with large sample size and robust methodology are warranted in future research.

## Figures and Tables

**Table 1:** Searching strategy examples.

Items	Searching strategy example
*PubMed*	
#1	(((acupuncture[MeSH terms]) OR acupuncture therapy[MeSH terms]) OR acupuncture points[MeSH terms]) OR acupressure[MeSH terms]
#2	((((acupunctur^*∗*^[Title/Abstract]) OR acupoin^*∗*^[Title/Abstract]) OR acupressur^*∗*^[Title/Abstract]) OR massag^*∗*^[Title/Abstract]) OR plaster^*∗*^[Title/Abstract]
#3	#1 OR #2
#4	(“neoplasms”[MeSH terms]) OR “cancer survivors”[MeSH terms]
#5	((((((neoplasm^*∗*^[Title/Abstract]) OR tumor^*∗*^[Title/Abstract]) OR tumour^*∗*^[Title/Abstract]) OR cancer^*∗*^[Title/Abstract]) OR malignan^*∗*^[Title/Abstract]) OR carcinoma^*∗*^[Title/Abstract]) OR oncolog^*∗*^[Title/Abstract]
#6	#4 OR #5
#7	((“sleep initiation and maintenance disorders”[MeSH terms])) OR “sleep wake disorders”[MeSH terms]
#8	(((Sleep^*∗*^[Title/Abstract]) OR insomnia“[Title/Abstract]) OR sleep disturbance^*∗*^[Title/Abstract]) OR sleep disorder^*∗*^[Title/Abstract]
#9	#7 OR #8
#10	#6 AND #9
#11	#3 AND #10
#12	(((((((((“randomized controlled trial”[Publication Type]) OR “controlled clinical trial”[Publication Type]) OR “ramdomized”[Title/Abstract]) OR “ramdomised”[Title/Abstract]) OR “placebo”[Title/Abstract]) OR “sham”[Title/Abstract]) OR “randomly”[Title/Abstract]) OR “trial”[Title/Abstract]) OR “groups”[Title/Abstract])
#13	#11 AND #12
#14	(animals[MeSH terms] NOT (humans[MeSH terms] AND animals[MeSH terms]))
#15	#13 NOT #14

*Cochrane Central Register of Controlled Trials (CENTRAL)*	
#1	MeSH descriptor: [acupuncture] explode all trees
#2	MeSH descriptor: [acupuncture therapy] explode all trees
#3	MeSH descriptor: [acupuncture points] explode all trees
#4	MeSH descriptor: [acupressure] explode all trees
#5	“acupunctur^*∗*^”:ti,ab, kw or acupoin^*∗*^:ti,ab, kw or acupressur^*∗*^:kw or massag^*∗*^:ti,ab, kw or plaster^*∗*^:ti,ab, kw (word variations have been searched)
#6	#1 or #2 or #3 or #4 or #5
#7	MeSH descriptor: [neoplasms] explode all trees
#8	MeSH descriptor: [cancer survivors] explode all trees
#9	neoplasm^*∗*^:ti,ab, kw or tumor^*∗*^:ti,ab, kw or tumour^*∗*^:ti,ab, kw or cancer^*∗*^:ti,ab, kw or malignan^*∗*^:ti,ab, kw or carcinoma^*∗*^:ti,ab, kw or oncolog^*∗*^:ti,ab, kw (word variations have been searched)
#10	#7 or #8 or #9
#11	#6 and #10
#12	MeSH descriptor: [sleep initiation and maintenance disorders] explode all trees
#13	MeSH descriptor: [sleep-wake disorders] explode all trees
#14	Sleep^*∗*^:ti,ab, kw or insomnia:ti,ab, kw or sleep disturbance^*∗*^:ti,ab, kw or sleep disorder^*∗*^:ti,ab, kw (word variations have been searched)
#15	#12 or #13 or #14
#16	#11 and #15
#17	Trials + Cochrane protocol

*Embase Classic* *+* *Embase*	
#1	Acupuncture^*∗*^.m_titl.
#2	Acupressur^*∗*^.m_titl.
#3	acupoin^*∗*^.m_titl.
#4	massag^*∗*^.m_titl.
#5	#1 or #2 or #3 or #4
#6	Neoplasm^*∗*^.mp. [mp = ti, ab, ot, nm, hw, kf, px, rx, ui, an, tx, sh, ct, sa, tn, dm, mf, dv, kw]
#7	“tumor^*∗*^”.m_titl.
#8	“tumour^*∗*^”.m_titl.
#9	cancer^*∗*^.mp. [mp = ti, ab, ot, nm, hw, kf, px, rx, ui, an, tx, sh, ct, sa, tn, dm, mf, dv, kw]
#10	malignan^*∗*^.mp. [mp = ti, ab, ot, nm, hw, kf, px, rx, ui, an, tx, sh, ct, sa, tn, dm, mf, dv, kw]
#11	carcinoma^*∗*^.mp. [mp = ti, ab, ot, nm, hw, kf, px, rx, ui, an, tx, sh, ct, sa, tn, dm, mf, dv, kw]
#12	oncolog^*∗*^.mp. [mp = ti, ab, ot, nm, hw, kf, px, rx, ui, an, tx, sh, ct, sa, tn, dm, mf, dv, kw]
#13	#6 or #7 or #8 or #9 or #10 or #11 or #12
#14	#5 and #13
#15	Sleep^*∗*^.mp. [mp = ti, ab, ot, nm, hw, kf, px, rx, ui, an, tx, sh, ct, sa, tn, dm, mf, dv, kw]
#16	Insomnia^*∗*^.mp. [mp = ti, ab, ot, nm, hw, kf, px, rx, ui, an, tx, sh, ct, sa, tn, dm, mf, dv, kw]
#17	sleep disturbance^*∗*^.m_titl.
#18	sleep disorder^*∗*^.m_titl.
#19	#15 or #16 or #17 or #18
#20	#14 and #19

*Wanfang Database*	
题名或关键词: ((“针灸” + “穴位按压” + “穴位按摩” + “穴位”) ^*∗*^ (“失眠” + “睡眠障碍” + “睡眠”) ^*∗*^ (“癌症” + “肿瘤”+ “癌性”))	

**Table 2:** Characteristics of RCTs examining somatic acupoint stimulation included in systematic review.

First author, year, setting	Study design	Participants (*n*)	SAS type	Intervention		Follow-up	Sleep-related outcomes
				Intervention group(s)	Sham/control group(s)		
H.T. Hoang, 2019, Vietnam [32]	Randomized controlled trial (registered ongoing/completed study)	Randomized = 114, 38 patients in each group	Self-administered acupressure	Self-acupressure group: standard care and true self-acupressure intervention protocol	Placebo comparator group: enhanced standard care + sham self-acupressure No intervention group: enhanced standard care	Weekly phone call follow-up (during the four-week intervention)	ISI and sleep parameter in sleep diary

S.M. Zick, 2016, USA [22]	Randomized controlled trial (published study)	Randomized = 288 Completed = 228 Relaxing acupressure group: 74 (completed) Stimulating acupressure group: 70 (completed) Usual care group: 84 (completed)	Self-administered acupressure	Relaxing acupressure (intervention 1), stimulating acupressure (intervention 2)	Usual care	4-week follow-up	PSQI
Y.L. Zhao, 2015, China [29]	Randomized controlled study (published study, thesis)	Randomized = 208 Completed = 190 Intervention group: 93 (completed) Control drug group: 97 (completed)	Acupuncture (including moxibustion as an additional treatment)	Acupuncture + moxibustion	Control drug: estazolam	7-day follow-up	AIS and PSQI

W.R. Tan, 2014, Taiwan [30]	Experimental pilot study, randomized controlled study (published study	Randomized = 57 Completed = 45 Acupressure/oils group: 15 (completed) Acupressure only group: 16 (completed) Control group: 14 (completed)	Self-administered acupressure	Acupressure + oils group: acupressure with 5% essential oil; acupressure includes kneading, pointing, pressing, and pushing. Acupressure only group: includes kneading, pointing, pressing, and pushing.	Sham acupressure	NR	PSQI

S. Bokmand, 2013, Denmark [19]	Randomized controlled trial (published study)	Randomized = 94 Completed = 94 Intervention group: 31 (completed) Sham group: 29 (completed) No-treatment group: 34 (completed)	Manual acupuncture	Manual acupuncture in the selected acupuncture points	Sham group: manual acupuncture in the selected sham points. No-treatment group: received no acupuncture.	NR	Disturbed night sleep (rated as “yes” or “no”)

Y. Feng, 2011, China [21]	Randomized controlled trial (published study)	Randomized = 80 Completed = 80 Intervention group: 40 (completed) Control group: 40 (completed)	Manual acupuncture	Manual acupuncture on the acupoints	Standard care with fluoxetine hydrochloride	NR	PSQI

S. David, 2010, USA [31]	Randomized controlled trial (registered ongoing/completed study)	Randomized = 65;	Acupuncture	Acupuncture	A validated sham acupuncture control	One month following the completion of intervention and six months after the conclusion of intervention	Insomnia reduction

SAS: somatic acupoint stimulation; ISI: Insomnia Severity Index; PSQI: The Pittsburgh Sleep Quality Index; AIS: Athens insomnia scale; NR: not reported.

**Table 3:** Somatic acupoint stimulation protocols used in the included studies.

First author, year, country	SAS type	SAS protocols			
		Selected acupoints: no. and names	Responses elicited	Instructions of SAS	Total sessions
H.T. Hoang, 2019, Vietnam [32]	Self-acupressure	6: Baihui (DU/GV 20), Yintang (EX-HN3), Fengchi (GB20), Neiguan (PC6), Shenmen (HT7), and Taichong (LR3)	NR	Daily acupressure at night with stimulation for each acupoint lasting 3 minutes, 24 minutes in total. Participants received a weekly phone call follow-up from the study investigator to remind them to self-practice acupressure at home. Training: participants received a self-acupressure training section at hospital from four trained nurses.	4 weeks (28 days, 28 sessions)

S.M. Zick, 2016, USA [22]	Self-administered acupressure	Relaxing acupressure: 5: *Yintang (*EX-HN3, centrally), *Anmian* (bilaterally), heart 7 (Shenmen, HT7, bilaterally), spleen 6 (Sanyinjiao, SP6, bilaterally), and liver 3 (Taichong, LR3, bilaterally). Stimulating acupressure: 6: Baihui (DU/GV 20, centrally), conception vessel 6 (Qihai, CV6, centrally), large intestine 4 (Hegu, LI14, bilaterally), stomach 36 (Zusanli, ST36, bilaterally), spleen 6 (Sanyinjiao, SP6, bilaterally), and kidney 3 (Taixi, KI3 bilaterally).	NR	Once per day. Participants were required to stimulate the acupoint in a circular motion with 3 minutes for each acupoint. Training: patients were taught to self-administer acupressure by a trained acupressure educator.	6 weeks (42 sessions)

Y.L. Zhao, 2015, China [29]	Acupuncture (including moxibustion)	Acupuncture: 6: Baihui (DU/GV20), Shenting (GV24), Yintang (EX-HN3), Shenmen (HT7), Zusanli (ST36, bilaterally), Sanyinjiao (SP6, bilaterally), and Guangyuan (CV4). Moxibustion: 2: Shenque (CV8) and Guangyuan (CV4).	*De qi*	Once per day for 7 days, 30 minutes each time (acupuncture + moxibustion). Needle type (depths of insertion): stainless steel (GV20 : 0.5–0.8 cun, GV24 : 0.5–0.8 cun, EX-HN3: 0.5–0.8 cun, HT7: 0.3–0.5 cun, ST36 : 1.2–1.5 cun, SP6: 1.0–1.2 cun, and CV4: 1.0–1.5 cun).	7 days (7 sessions)

W.R. Tan, 2014, Taiwan [30]	Self-administered acupressure	3: Hegu (LI4, bilaterally), Zusanli (ST36, bilaterally), and Sanyinjiao (SP6, bilaterally). Acupressure with essential oils (group A): essential oils were used for each acupoint acupressure every time.	*De qi*	Daily application (6 min) on each morning at the chosen acupoints, and each acupoint should be pressed in rotation for 1 minute. Acupressure skills consist of pointing, pressing, kneading, and pushing.	5 months (around 150 sessions)

S. Bokmand, 2013, Denmark [19]	Manual acupuncture	4: Neiguan (HC6, bilaterally), Taixi (KI3, bilaterally), Sanyinjiao (SP6, bilaterally), and Taichong (LR3, bilaterally)	NR	One treatment per week for 15–20 minutes. Needle type (depths of insertion): NR (NR).	5 weeks (5 sessions)

Y. Feng, 2011, China [21]	Manual acupuncture	9: Baihui (DU/GV 20), Fenglong (ST 40), Neiguan (PC 6), Sanyinjiao (SP 6), Sishencong (EX-HN1), Shenmen (TF 4), Yinlingquan (SP 9), Xuehai (SP 10), and Yintang (EX-HN3).	NR	Daily treatment for 20–30 min. Needling manipulation performed by the acupuncturist at the interval of 5–10 minutes. Needle type (depths of insertion): NR (NR).	30 days (30 sessions)

S. David, 2010, USA [31]	Acupuncture	NR	NR	Approximately 20 minutes	10 sessions

NR: not reported.

**Table 4:** Methodological quality assessment of included trials.

	Trials	Item 1	Item 2	Item 3	Item 4	Item 5	Item 6	Item 7	Item 8	Item 9	Item 10	Item 11	Item 12	Item 13	Total
1	H.T. Hoang, 2019, Vietnam [32]	Yes	Yes	Yes^*∗*^	No	Yes^*∗*^	NA	NA	NA	NA	Yes	NA	Yes	Unclear	6

2	S.M. Zick, 2016, USA [22]	Yes	Yes	Yes	No	Yes	Yes	Yes	Yes	Yes	Yes	Yes	Yes	Yes	12

3	Y.L. Zhao, 2015, China [29]	Yes	Yes	No	No	Yes	Yes	Yes	No	Yes	Unsure	Yes	Yes	Unsure	8

4	W.R. Tan, 2014, Taiwan [30]	Yes	No	Yes	No	Yes	Yes	Yes	Yes	Yes	Yes	Yes	Yes	Yes	11

5	S. Bokmand, 2013, Denmark [19]	Yes	Yes	Yes^*∗∗*^	Yes^*∗∗*^	Yes^*∗∗*^	Yes	Yes	Yes	Yes	No	Yes	Yes	Unsure	11

6	Y. Feng, 2011, China [21]	Yes	Unsure	Unsure	Unsure	Unsure	Yes	Yes	Unsure	Yes	Unsure	Unsure	Yes	Unsure	5

7	S. David, 2010, USA [31]	Unsure	Unsure	No	No	No	NA	NA	NA	NA	Unsure	NA	Yes	Unsure	1

*Item 1.* “Was the method of randomization adequate?” *Item 2.* “Was the treatment allocation concealed?” *Item 3.* “Was the patient blinded to the intervention?” *Item 4.* “Was the care provider blinded to the intervention?” *Item 5.* “Was the outcome assessor blinded to the intervention?” *Item 6.* “Was the drop-out rate described and acceptable?” *Item 7.* “Were all randomized participants analyzed in the group to which they were allocated?” *Item 8.* “Are reports of the study free of suggestion of selective outcome reporting?” *Item 9.* “Were the groups similar at baseline regarding the most important prognostic indicators?” *Item 10* “Were cointerventions avoided or similar?” *Item 11* “Was the compliance acceptable in all groups?” *Item 12* “Was the timing of the outcome assessment similar in all groups?” *Item 13.* “Are other sources of potential bias unlikely?—*Source*: A. D. Furlan, A. Malmivaara, R. Chou, C. G. Maher, R. A. Deyo, M. Schoene, . . . M. W. Van Tulder (2015). 2015 updated method guideline for systematic reviews in the Cochrane Back and Neck Group. Spine, 40(21), 1660-1673. NA: not applicable due to the nature of registered ongoing/completed trials without publishing the final results. ^*∗*^The trial investigator stated that a partial blinding design was utilized for participants in the true and sham acupressure groups. Partial blinding design was also used for the true and sham intervention groups given the outcome assessors were the study participants themselves. ^*∗∗*^The clinical study authors stated that acupuncture group and sham acupuncture group were both patient- and investigator-blinded. Subjective symptoms were self-assessed and the outcome assessors were therefore deemed as the participants themselves.
